# Neoadjuvant stereotactic radiosurgery for intracerebral metastases of solid tumors (NepoMUC): a phase I dose escalation trial

**DOI:** 10.1186/s40880-019-0416-2

**Published:** 2019-11-09

**Authors:** Christian D. Diehl, Ehab Shiban, Christoph Straube, Jens Gempt, Jan J. Wilkens, Markus Oechsner, Carmen Kessel, Claus Zimmer, Benedict Wiestler, Bernhard Meyer, Stephanie E. Combs

**Affiliations:** 1 Department of Radiation Oncology, Klinikum rechts der Isar Hospital, Technical University of Munich, Ismaninger Straße 22, 81675 Munich, Germany; 2 Department of Radiation Sciences, Institute of Innovative Radiotherapy, 85764 Neuherberg, Germany; 3 Department of Neurosurgery, Klinikum rechts der Isar Hospital, Technical University of Munich, 81675 Munich, Germany; 4 Department of Diagnostic and Interventional Neuroradiology, Klinikum rechts der Isar Hospital, Technical University of Munich, 81675 Munich, Germany

**Keywords:** Cancer, Intracerebral metastasis, Stereotactic radiosurgery, Fractionated stereotactic radiation therapy, Microsurgical resection, Neoadjuvant radiation therapy, Maximum tolerated dose, Protocol

## Abstract

**Background:**

More than 25% of patients with solid cancers develop intracerebral metastases. Aside of surgery, radiation therapy (RT) is a mainstay in the treatment of intracerebral metastases. Postoperative fractionated stereotactic RT (FSRT) to the resection cavity of intracerebral metastases is a treatment of choice to reduce the risk of local recurrence. However, FSRT has to be delayed until a sufficient wound healing is attained; hence systemic therapy might be postponed. Neoadjuvant stereotactic radiosurgery (SRS) might offer advantages over adjuvant FSRT in terms of better target delineation and an earlier start of systemic chemotherapy. Here, we conducted a study to find the maximum tolerated dose (MTD) of neoadjuvant SRS for intracerebral metastases.

**Methods:**

This is a single-center, phase I dose escalation study on neoadjuvant SRS for intracerebral metastases that will be conducted at the Klinikum rechts der Isar Hospital, Technical University of Munich. The rule-based traditional 3 + 3 design for this trial with 3 dose levels and 4 different cohorts depending on lesion size will be applied. The primary endpoint is the MTD for which no dose-limiting toxicities (DLT) occur. The adverse events of each participant will be evaluated according to the Common Terminology Criteria for Adverse Events (CTCAE) version 5.0 continuously during the study until the first follow-up visit (4–6 weeks after surgery). Secondary endpoints include local control rate, survival, immunological tumor characteristics, quality of life (QoL), CTCAE grade of late clinical, neurological, and neurocognitive toxicities. In addition to the intracerebral metastasis which is treated with neoadjuvant SRS and resection up to four additional intracerebral metastases can be treated with definitive SRS. Depending on the occurrence of DLT up to 72 patients will be enrolled. The recruitment phase will last for 24 months.

**Discussion:**

Neoadjuvant SRS for intracerebral metastases offers potential advantages over postoperative SRS to the resection cavity, such as better target volume definition with subsequent higher efficiency of eliminating tumor cells, and lower damage to surrounding healthy tissue, and much-needed systemic chemotherapy could be initiated more rapidly.

*Trial registration* The local ethical review committee of Technical University of Munich (199/18S) approved this study on September 05, 2018. This trial was registered on German Clinical Trials Register (DRKS00016613; https://www.drks.de/drks_web/navigate.do?navigationId=trial.HTML&TRIAL_ID=DRKS00016613) on January 29, 2019.

## Background

Intracerebral metastases are a frequent cause of oncological morbidity and mortality that affect up to 25% of cancer patients; in two thirds of these patients, intracerebral metastasis originates from lung cancer, breast cancer, and malignant melanoma [1–4]. With the introduction of new systemic therapies, a better extracranial tumor control and hence longer survival have been achieved. However, since those new agents barely cross the brain–blood barrier, the incidence of intracerebral metastasis has not been favorably affected [5, 6]. With novel treatment strategies and hence prolonged survival, there is a high demand on safe local strategies with minimal interruption time or delay of systemic treatments. Therefore, efficient management of intracerebral metastasis is a challenge.

Up to date, neurosurgical resection, stereotactic radiosurgery (SRS), fractionated stereotactic radiation therapy (FSRT), and whole-brain radiation therapy (WBRT) are the main treatment modalities for intracerebral metastases [7]. Surgical resection is an effective treatment aiming to relieve symptoms associated with mass pressure by the tumor or surrounding edema. In case of unknown primary resection is mandatory to gain tissue for pathologic analysis. Overall, a patient’s sufficient physical condition is mandatory to undergo such an invasive treatment [8]. First data of the pre-magnetic resonance imaging (MRI) era even suggested an increased overall survival (OS) for patients who underwent the resection of solitary intracerebral metastases [9]. Adversely, about 46%–59% of patients will have a local recurrence due to remnant tumor cells after resection of intracerebral metastases [10, 11]. Over the last decades, several studies have shown that the combination of microsurgical resection followed by WBRT led to lower local and distant recurrence rates compared to surgical resection alone [11, 12]. However, WBRT is also strongly associated with neurocognitive function decline and impaired quality of life (QoL) [13, 14]. Therefore, postoperative radiotherapy (RT) to the resection cavity is now considered to be a treatment of choice, and this technique has shown a superior local control as compared with a surveillance strategy as well as an improved neurocognitive safety profile when compared with WBRT [15, 16]. Despite a lack of comparative evidence, fractionated RT has been shown to be equally efficient, with a convincing safety profile [17, 18]. In case of local or distant recurrence, salvage therapies such as WBRT, SRS, FSRT, and microsurgical resection can be performed [19]. Postoperative FSRT can be initiated after adequate wound healing and is applied to the tumor bed including potential tumor remnants with an extra margin of 1–5 mm to cover microscopic spread and to compensate setup inaccuracies. It has to be taken in account that the resection cavities tend to shrink within a few days after surgery. Therefore, a timely MRI is obligatory for treatment planning [20]. Lately, several studies have focused on neoadjuvant SRS before resection of intracerebral metastases for better target delineation and hence better sparing of surrounding tissue [21–23]. Notably, the inclusion of the surgical tract into the clinical target volume (CTV) is currently recommended by the American Society of Radiation Oncology (ASTRO) guidelines, and neoadjuvant SRS consequently reduces CTV [24].

This trial aims to escalate the dose for neoadjuvant SRS up to the dose thresholds depending on tumor size recommended by the German expert group on stereotactic radiation oncology [25]. Given the improved efficacy of systemic cancer therapies, long-lasting local control becomes of growing importance. Since local FSRT aims to prolong the time interval until the application of WBRT, locally efficient doses have to be applied.

## Methods

### Ethical approval, information, and safety

The experimental setup was approved by the local ethics committee of Technical University of Munich (Registration Number: 199/18S; München, Germany) and conducted in accordance with the Declaration of Helsinki and the Principles of the Good Clinical Practice Guidelines. The regulations regarding medical confidentiality and data protection are fulfilled. A submission to the Bundesamt für Strahlenschutz is not required this was confirmed by the expert commission of the German Society of Radiation Oncology (DEGRO, No. 141) (Additional file 1). The latest version of the protocol is NepoMUC Clinical Trial Protocol Version 1.1, Date August 08, 2018. The SPIRIT© (http://www.spirit-statement.org) checklist was applied for this study protocol.

Informed consent will be obtained from all participants. The participants will be informed about the General Data Protection Regulation (EU) 2016/679 in the informed consent form.

### Data collection, management, and analysis

As used in this study, the term Case Report Form (CRF) should be understood to refer to a paper form or an electronic data record or both, depending on the data collection method used in this trial. For this trial, relevant data will be documented in paper-printed CRFs. All findings including clinical and laboratory data will be documented in the subject’s medical record and in the CRF by the investigator or an authorized member of the study team. The investigator is responsible for ensuring that all sections of the CRF are completed correctly and that entries can be verified against source data. In some cases, the CRF, or part of the CRF, may also serve as source documents: Karnofsky Performance Status, Documentation of Clinical-Neurological Examination. In these cases, a document should be available at the investigator’s site and clearly identify those data that will be recorded in the CRF, and for which the CRF will stand as the source document. There is no financial compensation for the participant. In case that the participant discontinues the study, documented medical parameters will not be collected and used for statistical analysis. In case of deviation from intervention protocol, e.g., no SRS prior to resection, the participant is excluded from the study.

### Data management

According to the §13 of the German Good Clinical Practice (GCP) Regulation, all important trial documents (e.g., CRF) will be archived for at least 10 years after the trial termination. According to the §28c of the German X-Ray Regulation and the §87 of the German Radiation Protection Regulation, the informed consent forms including patients’ consent for trial participation, application of irradiation, and data transmission to the competent authority will be archived for at least 30 years after the trial termination. The study center at the Department of Radiation Oncology will be responsible for archiving the trial master file (TMF) including protocol, CRF, report, and so on. The investigator(s) will archive all trial data [data and Investigator Site File (ISF)] including subject identification list and relevant correspondence) according to the section 4.9 of the International Council for Harmonisation of Technical Requirements for Registration of Pharmaceuticals for Human Use (ICH) Consolidated Guideline on GCP (E6) and to local law or regulations. The Subject Identification List will be archived for at least 15 years after the trial termination. If an investigator relocates, retires, or for any reason withdraws from the study, the principal investigator should be notified prospectively. The study records must be transferred to an acceptable designee, such as another investigator or another institution.

### Study design

The trial is designed as a single-center, dose escalation study. Patients fulfilling the inclusion and exclusion criteria will be allocated to neoadjuvant SRS and assigned to four different cohorts depending on tumor size. Within each cohort, there are 3 different dose levels. The treatment contains neoadjuvant SRS according to the protocol, tumor resection according to imaging findings, and postoperative neuropathological evaluation.

### Objectives

The primary objective is to determine the maximum tolerated dose (MTD). Secondary objectives are to evaluate further parameters such as survival and tumor characteristics, assessment of QoL, clinical neurological and neurocognitive functions.

### Endpoints

The primary endpoint is the MTD for which no dose-limiting toxicities (DLTs) occur. DLTs will be evaluated according to the Common Terminology Criteria for Adverse Events (CTCAE) version 5.0 (2017) continuously during the study until the first follow-up visit (4–6 weeks after surgery) for each patient and each dose level. Central nerve system necrosis (≥ grade 3), cerebrospinal fluid leakage (≥ grade 4), wound infection ((≥ grade 4), wound dehiscence (≥ grade 4), postoperative hemorrhage (≥ grade 4), cognitive disturbance (≥ grade 4), cerebral edema (≥ grade 4), headache (≥ grade 4), and seizure (≥ grade 4) are defined as DLTs.

Secondary endpoints are as follows:

- Central nerve system necrosis of CTCAE grade 1–3.
- Cerebrospinal fluid leakage of CTCAE grade 1–3.
- Wound infection of CTCAE grade 1–3.
- Wound dehiscence of CTCAE grade 1–3.
- Cerebral edema of CTCAE grade 1–3.
- Local control rates.
- OS and progression-free survival (PFS) at 12-month follow-up.
- Time interval between treatment initiation of neoadjuvant SRS and start of systemic chemotherapy.
- Health-related QoL evaluated using the EuroQoL questionnaire.
- Neurocognitive function assessed using the Minimal Mental State Examination (MMSE) test.
- Late toxicity of CTCAE grade 1–5.
- Immunological and molecular parameters [such as programmed death-ligand 1 (PD-L1) expression, Rapidly Accelerated Fibrosarcoma type B (BRAF) gene mutation, epidermal growth factor receptor (EGRF) mutation, and anaplastic lymphoma kinase [ALK] translocation on the resected specimen].

### Patient selection

Patients with the diagnosis of intracerebral metastasis on contrast-enhanced MRI will be evaluated and screened for the protocol. All patients fulfilling the inclusion and exclusion criteria will be informed about the study. Registration for the study must be performed prior to the initiation of RT. The study treatment (neoadjuvant SRS and/or neurosurgical intervention) should be initiated no later than 3 weeks after diagnosis of intracerebral metastases.

Patients meeting all of the following inclusion criteria will be considered for admission to the trial:

- Patients with 1–4 intracerebral metastases observed on contrast-enhanced MRI from histologically confirmed solid tumors.
- One intracerebral metastasis is ≥ 3 cm in diameter or
  - Persisting neurologic symptoms or symptomatic epilepsy from intracerebral metastases despite treatment with steroids.
  - Tumor location close to eloquent brain areas therefore neurological symptoms can be expected without long-term steroidal medication.
  - Patient decides to undergo surgical intervention, if resection and radiotherapy are equal treatment options or if the patient declines radiotherapy.
- Age ≥ 18 years of age.
- Karnofsky performance score ≥ 70, Eastern Cooperative Oncology Group (ECOG) performance score ≤ 1.
- Women with childbearing potential should have adequate contraception.
- Ability of subject to understand character and individual consequences of the clinical trial.
- Written informed consent (must be available before enrolment in the trial).

Patients presenting with any of the following exclusion criteria will not be included in the trial:

- Patients with unknown primary tumor.
- The diameter of any single lesion exceeding 4 cm.
- Tumors causing severe neurological deficits or with mass effect requiring immediate surgical intervention.
- Previous radiotherapy to the brain.
- Known histological type of small cell cancer, germ cell cancer, or lymphoma.
- Patient refuses to take part in the study.
- Patients who have not yet recovered from acute toxicities of prior therapies.
- Clinically active renal, liver, or cardiac disease.
- Known carcinoma within 5 years (excluding carcinoma in situ of the cervix, basal cell carcinoma, squamous cell carcinoma of the skin) requiring immediate treatment that interferes with the study therapy.

### Radiotherapy

#### Treatment planning

All patients will be prepared for highly advanced SRS. Individual mask fixation will be performed for each patient. Computed tomography (CT) with and without contrast enhancement will be performed with the patient in individual mask fixation. Additionally, MRI with and without contrast enhancement will be performed for target volume delineation.

#### Target volume delineation for neoadjuvant SRS

Gross target volume (GTV)—macroscopic lesion visible on MRI with T1-weighted contrast enhancement.

Clinical target volume (CTV)—GTV plus a safety margin of 2 mm accounting for microscopic spread.

Planning target volume (PTV)—CTV plus a margin of 1–3 mm accounting for movement and positioning inaccuracies.

#### Dose prescription

Neoadjuvant SRS is applied in a single fraction with single doses depending on the volume and location of the intracerebral metastases requiring neurosurgical resection. The starting dose for the dose escalation scheme will be the dose used by Asher et al. [21] that was already proven to be safe and tolerable in a neoadjuvant setting. The dose will be increased by 2 Gy increments up to the dose recommended by the DEGRO working group on SRS for intracerebral metastases (Table 1) [25]. Doses are prescribed to the 80% isodose line using a linear accelerator.

**Table 1:** Cohorts and neoadjuvant SRS dose levels in the present study

Cohort	SRS dose level (Gy)		
	I	II	III
Lesion of ≤ 1.0 cm	18	20	22
Lesion of 1.1–2.5 cm	16	18	20
Lesion of 2.6–3.0 cm	14	16	18
Lesion of 3.1–4.0 cm	12	14	16

The patients will be assigned to 4 different cohorts depending on the largest diameter of the intracerebral metastases to be radiosurgically treated before surgical removal. Each cohort has 3 dose levels. Size and dose were chosen according to the recommondations of the German Society of Radiation Oncology (DEGRO) working group on SRS for intracerebral metastases. The first 3 patients in the first cohort will be treated at a starting dose. If no dose-limiting toxicity (DLT) occurs in that dose level, the following 3 patients will be treated at the next higher dose level; if any of the first 3 patients experiences a DLT, the following 3 patients will be treated at the same dose level. At the highest dose level, at least 6 patients will be treated. Dose will be prescribed to the 80% isodose for linear accelerator (LINAC)-based radiotherapy

*SRS* stereotactic radiosurgery

If further metastases are present (up to 4 in total, no lesion exceeding 4 cm in diameter) that do not require resection, those lesions will be treated with SRS according to the guidelines of the DEGRO working group on SRS for intracerebral metastases [25]. Quantitative analysis of normal tissue effects in the clinic (QUANTEC) reports are applied for dose constraints of normal tissues [26, 27].

### Neurosurgery

For optimal surgical planning, all patients will have an MRI for navigation purposes. For eloquent lesions (less than 2 cm distance between the metastases and the corticospinal tract or the Broca’s area), intraoperative neuromonitoring utilizing direct cortical and subcortical electrical stimulation and navigated transcranial magnetic stimulation will be performed to minimize the risk of new neurological deficit [28–31]. Tumor resection will be carried out according to microsurgical standards. Surgical parameters, such as estimated blood loss, duration of surgery, need for blood transfusions, and complications will be documented in a standardized fashion. To reflect the daily practice and not the best surgical approach for individual cases, there will be no mandate on a specific surgical approach, and each center will decide the surgical approach independently. For the same reasons, individual surgical experience cannot be taken into consideration, but experienced centers have been selected. Postoperative MRI for resection control will be done in all patients, thereby special emphases will also be drawn for postoperative ischemia. Postoperative care is not standardized in the study protocol. The use of analgesics and cortisol follows the local routine, but needs to be documented.

### Sample size calculation

Sample size was determined using the traditional “3 + 3” design as explained below. As radiation dose will differ according to the lesion size, 4 independent cohorts of patients will be evaluated for each lesion size/dose level. With 3 dose levels in 4 different cohorts according to the lesion size, this will lead to a minimal sample size of 4 × [(3 × 3) + 3] = 48 patients, assuming that at least 6 patients should have been tested at MTD in each cohort and that no DLTs occur. In case of DLTs in any patient of a certain dose level group, the size of this group will be enlarged to 6 patients. Therefore, the final sample size might vary.

### Statistical analyses

The traditional “3 + 3” design remains the prevailing method for conducting phase I cancer clinical trials [32, 33]. It requires no modelling of the dose-toxicity curve beyond the classical assumption for cytotoxic drugs that toxicity increases with dose. This rule-based design proceeds with cohorts of 3 patients. The first 3 patients in the first cohort will be treated at a starting dose that is considered to be safe based on extrapolation. If none experiences a TLD at that dose level, the following 3 patients will be treated at the next higher dose level. The MTD for which no DLT occur will be evaluated according to the CTCAE Version 5.0 (2017) continuously during the study until first follow up (4–6 weeks after surgery) for each cohort and dose level. A DLT is defined as central nerve system necrosis (≥ grade 3) or cerebrospinal fluid leakage (≥ grade 4) or wound infection (≥ grade 4) or wound dehiscence (≥ grade 4) or postoperative hemorrhage (≥ grade 4) or cognitive disturbance (≥ grade 4) or cerebral edema (≥ grade 4) or headache (≥ grade 4) or seizure (≥ grade 4). If no DLT occurs in one dose level, another 3 patients will be treated at the next higher dose level. However, if any of the first 3 patients experiences a DLT, the following 3 patients will be treated at the same dose level. At the highest dose level, at least 6 patients will be treated. Thus, the maximum number of patients enrolled in this study will be 4 × 6 × 3 = 72. Dose will be prescribed to the 80% isodose for linear accelerator (LINAC)-based radiotherapy. The dose escalation continues until at least 2 patients in a cohort of 3–6 patients experience DLTs (i.e., 33% of patients with DLTs at that dose level). The recommended dose for a future phase II trials is conventionally defined as the dose level just below the toxic dose level.

The primary endpoint of this study is safety assessed from the start of radiotherapy until the first follow up after surgery (4–6 weeks after surgery). Based on experience from clinical practice, the number of patients lost to follow-up is expected to be very small. Patients who dropped out of the study between radiation treatment and first follow-up visit will be replaced by a new patient. For patients without a final examination after 1 year, the last valid examination will be used for the assessment of secondary endpoints. All secondary endpoints will be analyzed in an explorative manner using appropriate statistical methods: Mann–Whitney *U* Test (acute toxicity of CTCAE grade 1–3; late toxicity of CTCAE grade 1–5, immunological parameters, time interval between treatment initiation and start of systemic chemotherapy), Fisher’s exact test or Chi-square test (local control rates), log-rank test and Kaplan–Meier curves (OS and PFS at 12-month follow-up), Student *t* test (neurocognitive function), and scaled *t* test (health-related QoL) for independent patients.

The following parameters will be collected and taken into account in analyses applying regression models: age, Karnofsky performance score, extent of neurosurgical resection, recursive partitioning analysis (RPA)-classification.

### Assessment of endpoints

The primary endpoint, MTD, will be assessed through extensive anamnesis and clinical neurological examination on the first postoperative day, the day of discharge from hospital, and the first follow-up visit at 4–6 weeks after surgery, with wound healing evaluated by an experienced neurosurgeon. This study will use the CTCAE version 5.0 for toxicity and adverse event reporting. Postoperative MRI (1–2 days after surgery) and the first follow-up MRI (4–6 weeks after surgery) will be evaluated by an experienced neuroradiologist, and the efficacy will be assessed according to the RECIST criteria [34].

For secondary endpoints, anamnesis and clinical neurological examination will be conducted as mentioned above. Disease progression is defined as radiological or neurological/clinical progression (whichever occurs first); PFS is considered the time interval between the date of treatment initiation and the date of disease progression or death, whichever occurs first. If neither event is observed, the patient will be censored at the date of the last follow-up examination. Neurocognitive function, QoL, and late toxicities will be evaluated at each follow-up visit every 3 months or at neurological deterioration (Table 2). Neuropathological work-up on the resected specimen will follow standard operating procedures at the Department of Pathology and will include immunologic as well as molecular parameters, such as PD-L1 expression, BRAF-gene mutation, EGFR mutation, and ALK translocation, where appropriate. Follow-up assessments (including MRI or CT) will be performed as described until disease progression (even after the termination of the study) in accordance with GCP and the treatment guidelines for patients with intracerebral metastases.

**Table 2:** Time schedule of this study

Timepoint	Enrollment	Treatment				Follow-up (months)				Close-out (months)
	Day 0	Preoperative RT Day 1	Resection Days 2–7	Postoperative Days 1–2	Postoperative Days 3–5	1,5	3	6	9	12
Enrollment										
Eligibility screening	X									
Informed consent	X									
Study visit	X	X			X	X	X	X	X	X
Planning CT	Within 1–7 days after enrollment									
Planning MRI	Within 1–7 days after enrollment									
Interventions										
Neoadjuvant SRS		X								
Resection			X							
Assessments										
MRI	X			X		X	X	X	X	X
Clinical-neurological examination	X	X	X	X	X	X	X	X	X	X
Minimental state examination	X					X	X	X	X	X
Health-related QoL	X				X	X	X	X	X	X
Toxicities	X				X	X	X	X	X	X
Adverse events		Continuous assessment				X	X	X	X	X

This table of study schedule is based on the the SPIRIT© checklist, minor changes were made for adaption to the NepoMUC protocol

*CT* computer tomography, *MRI* magnetic resonance tomography, *SRS* stereotactic radiosurgery

### Monitoring

An independent Data and Safety Monitoring Board (DSMB)/Data Monitoring Committee (DMC) will monitor the patient recruitment, reported adverse events, and data quality at least twice a year. Based on its review, the DSMB will provide the principal investigators (PIs) with recommendations regarding trial modification, continuation, or termination. The DSMB will be composed of independent experts in the field of radiation oncology. The mission of the DSMB will be to ensure the ethical conduct of the trial and to protect the safety interests of patients in this trial. Identified problems will be discussed with the PIs who will take appropriate measures. Relevant information (including relevant safety data) will be included in the study status reports, which serve as a basis of discussion for the study group meetings including the PI, study coordinator, and sub-investigators. The auditing will be conducted by the clinical site or by the DSMB and is independent from investigators and sponsors.

## Discussion

A study published by Asher et al. [21] evaluated the role of neoadjuvant SRS in 47 patients with 51 intracerebral metastasis lesions undergoing surgery at a median of 1 day (range 0–7 days) after neoadjuvant SRS. The median diameter of metastatic lesions was 3.0 cm (range 1.3–5.2 cm), and a dose reduction was applied with a median dose of 14.0 Gy (range 11.6–18.0 Gy) prescribed to the 80% isodose level. Local control rates were 97.8%, 85.6%, and 71.8% at 6, 12, and 24 months, respectively. Eight patients with local failure were re-operated and proved for recurrence without radiation necrosis. Local failure was more likely for lesions larger than 3.4 cm (*P* = 0.014). Due to the explorative character of their study, Asher et al. [21] were rather conservative in considering their dose prescription. Their doses were well below the dose thresholds that were established by the Radiation Therapy Oncology Group (RTOG) Trial 90-05, and it has to be kept in mind that those dose thresholds were set up for patients who had already received prior WBRT with a minimum dose of 30 Gy [35]. Patel et al. [22] conducted a trial, investigating neoadjuvant and postoperative SRS to the resection cavity in 180 patients with 189 intracerebral metastasis lesions being resected. In the neoadjuvant SRS cohort, the marginal dose was reduced by 20% (median dose, 14.5 Gy vs. 18.0 Gy) in analogy to that in the RTOG Trial 90-05 [35] with no extra margin added to the GTV (GTV = PTV) compared to the postoperative SRS cohort with an extra margin of 2 mm. GTV was similar, with 8.3 mL (range 0.89–46.8 mL) in the neoadjuvant SRS cohort versus 9.24 mL (range 0.68–54.60 mL) in the postoperative SRS cohort (*P* = 0.85). In the neoadjuvant cohort patients underwent intracerebral metastasis resection within 48 h after SRS. Outcomes were similar in regard of local recurrence, distant brain recurrence, and overall survival, but with significant lower rates of symptomatic radiation necrosis and leptomeningeal spread in the neoadjuvant SRS cohort than in the postoperative SRS cohort (4.9% vs. 16.4%, *P* = 0.01; 3.2% vs. 16.6%, *P* = 0.01) at 2 years, respectively [22]. In another work by Patel et al. [36], neoadjuvant SRS (66 patients with 71 lesions) was compared with postoperative WBRT (36 patients with 42 lesions); in analogy to that in the aforementioned study, the dose was reduced by 20% with no extra margin for PTV with surgery performed within 48 h after neoadjuvant SRS. Again, outcomes of the two cohorts were similar in terms of local recurrence, distant brain failure, and leptomeningeal disease recurrence. The rate of symptomatic radiation necrosis was higher in the neoadjuvant SRS cohort (5.6% vs. 0%), and the cavity size was significantly smaller (8.3 mL vs. 15.3 mL, *P* < 0.01) in this cohort. There was no analysis for QoL [36].

Vetlova et al. [23] analyzed a cohort of 19 patients with 22 metastases who underwent neoadjuvant SRS. The median tumor volume was 14.1 mL (range 3.0–57.1 mL), and the applied dose was 18 Gy in median (range 12.6–24.4 Gy), the surgery was carried out within 24–48 h after SRS. Two patients had local recurrence after 5.5 and 17.4 months of follow-up, and 1 had radiation necrosis at 4.6 months after treatment. Two patients died of disease progression.

The concept of neoadjuvant SRS for intracerebral metastases is characterized by a number of potential benefits compared with postoperative radiosurgery. Mostly, RT of the intact intracerebral metastases and surrounding normal tissues leads to a better definition of the target volume for RT, because postoperative changes, such as ischemia, scar tissue or blood-remnants, are missing. Therefore it is easier to spare normal tissue and consequently it can result in a higher safety of effective treatment of all tumor cells and subsequent lower damage to surrounding normal tissues. Additionally, the surgical tract does not exist before surgery; therefore, this area can be spared completely. Thereby, the rate of postoperative complications such as wound healing disorders and cerebrospinal fluid leaks could be reduced. After preoperative RT systemic chemotherapy or targeted therapy can be rapidly initiated after wound healing if needed in case of a high extracranial tumor burden. Contrary, systemic therapy is often delayed until postoperative RT is finished since some cancer therapies may not be applied during RT of the central nervous system or with high single doses.

This phase I study aims to find an optimal dose for neoadjuvant SRS for safe surgery and hence good outcome and local control. The trial incorporates a margin accounting for the microscopic spread and focuses on the dose escalation of this treatment strategy as well as QoL and neurocognitive function.

## Supplementary information

**Additional file 1.** Confrmation by the expert commission of the German Society of Radiation Oncology (DEGRO, No. 141) that a submission to the Bundesamt für Strahlenschutz is not required.

## Data Availability

The datasets collected and analyzed during the current study are available from the corresponding author on reasonable request.

## Abbreviations

SRS: stereotactic radiosurgery
FSRT: fractionated stereotactic radiosurgery
WBRT: whole-brain radiotherapy
MRI: magnetic resonance imaging
LINAC: linear accelerator
CTV: clinical target volume
ASTRO: American Society of Radiation Oncology
RT: radiation therapy
DEGRO: German Society of Radiation Oncology
CRF: Case Report Form
TMF: trial master file
ISF: Investigator Site File
ICH: International Council for Harmonisation
GCP: guideline on good clinical practice
MTD: maximum tolerated dose
DLT: dose limiting toxicity
CTCAE: Common Terminology Criteria for Adverse Events
MMSE: Minimal Mental State Examination
PD-L1: programmed death-ligand 1
ECOG: Eastern Cooperative Oncology Group
CT: computed tomography
GTV: gross tumor volume
CTV: clinical target volume
PTV: planning target volume
QUANTEC: quantitative analysis of normal tissue effects in the clinic
RPA: recursive partitioning analysis
PFS: progression-free survival
BRAF: B-Rapidly Accelerated Fibrosarcoma
EGFR: epidermal growth factor receptor
ALK: anaplastic lymphatic kinase
DSMB: Data and Safety Monitoring Board
DMC: monitoring committee
PI: principal investigator
RTOG: Radiation Therapy Oncology Group
