# Supplementary material for: Neoadjuvant stereotactic radiosurgery for intracerebral metastases of solid tumors (NepoMUC): a phase I dose escalation trial
Source: Cancer Commun (Lond). 2019 Nov 9;39:73. doi: 10.1186/s40880-019-0416-2 (PMC6842524; doi:10.1186/s40880-019-0416-2)
Supplement: Supplementary file 1 — Additional file 1. Confrmation by the expert commission of the German Society of Radiation Oncology (DEGRO, No. 141) that a submission to the Bundesamt für Strahlenschutz is not required. [file 40880_2019_416_MOESM1_ESM.pdf]

DEGRO DEUTSCHE GESELLSCHAFT FÜR RADIOONKOLOGIE E.V.

DEGRO • Reinhardtstraße 47 • 10117 Berlin

PD Dr. D. Habermehl
Klinik und Poliklinik für Radioonkologie und Strahlentherapie
Klinikum rechts der Isar
Technische Universität München
Ismaninger Str. 22
D - 81675 München

DEGRO GESCHÄFTSSTELLE
Reinhardtstraße 47
10117 Berlin
+49 30 8441 9188
+49 30 8441 9189
office@degro.org
www.degro.org

Entscheidung des „Unabhängigen Expertengremiums der DEGRO"[1]
gemäß Festlegung im Fachgespräch mit dem BfS vom 06.03.2003 und 05.06.2003

Die von

PD Dr. D. Habermehl, TU München

eingereichte Studie

„Neoadjuvante Radiotherapy (N-RT) for intracerebral Metastases of solid tumors NEPO_MUC"
(Anfrage 141)

wird von dem Expertengremium nach Ablauf der Bewertungsfrist einheitlich mit (3) Stimmen

als

[X] therapeutische Strahlenanwendung im Rahmen der Heilkunde

[ ] genehmigungsbedürftige Forschung nach §23 StrlSchV oder § 28a RöV

eingeschätzt.

Für das Expertengremium

22.01.2018

Prof. Dr. F. Wenz

Geschäftsführender Vorsitzender

[1] Mitglieder: Prof. Dr. F. Wenz (Geschäftsführender Vorsitz)
Prof. Dr. A. Grosu
Prof. Dr. W. Budach
Prof. Dr. M. Flentje
Prof. Dr. S. Combs
Prof. Dr. R. Sauer

Präsident: Prof. Dr. Dr. Jürgen Debus, Heidelberg • **Geschäftsführer:** Prof. Dr. Normann Willich, Münster
Vereinsregister Berlin 28605 B • Steuernummer: 27/640/57459
Deutsche Bank Privat- und Geschäftskunden AG Tübingen • IBAN DE94 6407 0024 0123 4111 00 • BIC DEUTDEDB640
